# Nasal Microbiome in COVID-19: A Potential Role of *Corynebacterium* in Anosmia

**DOI:** 10.1007/s00284-022-03106-x

**Published:** 2022-12-30

**Authors:** Carmela Nardelli, Giovanni Luca Scaglione, Domenico Testa, Mario Setaro, Filippo Russo, Carmela Di Domenico, Lidia Atripaldi, Massimo Zollo, Federica Corrado, Paola Salvatore, Biagio Pinchera, Ivan Gentile, Ettore Capoluongo

**Affiliations:** 1 Department of Molecular Medicine and Medical Biotechnologies, University of Naples Federico II, Naples, Italy; 2 CEINGE Biotecnologie Avanzate S.C.a R.L., Naples, Italy; 3 Task Force On Microbiome Studies, University of Naples Federico II, Naples, Italy; 4 Istituto Dermopatico Dell’Immacolata IDI-IRCSS, Rome, Italy; 5 Department of Otorhinolaryngology, Luigi Vanvitelli University of Naples, Naples, Italy; 6 Clinical Biochemistry Unit, AORN Ospedale Dei Colli, Naples, Italy; 7 Istituto Zooprofilattico Sperimentale del Mezzogiorno, Portici, Naples, Italy; 8 Department of Clinical Medicine and Surgery, University of Naples Federico II, Naples, Italy; 9 Department of Clinical Pathology and Genomics, Azienda Ospedaliera Per L’Emergenza Cannizzaro, Catania, Italy

## Abstract

The evolution and the development of the symptoms of Coronavirus disease 19 (COVID-19) are due to different factors, where the microbiome plays a relevant role. The possible relationships between the gut, lung, nasopharyngeal, and oral microbiome with COVID-19 have been investigated. We analyzed the nasal microbiome of both positive and negative SARS-CoV-2 individuals, showing differences in terms of bacterial composition in this niche of respiratory tract. The microbiota solution A (Arrow Diagnostics) was used to cover the hypervariable V1–V3 regions of the bacterial 16S rRNA gene. MicrobAT Suite and MicrobiomeAnalyst program were used to identify the operational taxonomic units (OTUs) and to perform the statistical analysis, respectively. The main taxa identified in nasal microbiome of COVID-19 patients and in Healthy Control subjects belonged to three distinct phyla: Proteobacteria (HC = 14%, Cov19 = 35.8%), Firmicutes (HC = 28.8%, Cov19 = 30.6%), and Actinobacteria (HC = 56.7%, Cov19 = 14.4%) with a relative abundance > 1% in all groups. A significant reduction of Actinobacteria in Cov19 group compared to controls (*P* < 0.001, FDR = 0.01) was found. The significant reduction of Actinobacteria was identified in all taxonomic levels down to the genus (*P* < 0.01) using the ANOVA test. Indeed, a significantly reduced relative abundance of *Corynebacterium* was found in the patients compared to healthy controls (*P* = 0.001). Reduced abundance of *Corynebacterium* has been widely associated with anosmia, a common symptom of COVID-19 as suffered from our patients. Contrastingly, the *Corynebacterium* genus was highly represented in the nasal mucosa of healthy subjects. Further investigations on larger cohorts are necessary to establish functional relationships between nasal microbiota content and clinical features of COVID-19.

**Supplementary Information:**

The online version contains supplementary material available at 10.1007/s00284-022-03106-x.

## Introduction

The WHO reported that pandemic Coronavirus Disease 2019 (COVID-19) has caused to date about six millions of deaths worldwide [1]. The severe acute respiratory syndrome coronavirus 2 (SARS-CoV-2) produces the following main symptoms: acute respiratory distress syndrome, fever, dry cough, tiredness, pain, nasal congestion, anosmia, sore throat, or gastrointestinal illness, such as diarrhea [2, 3]. Recently, many studies investigated possible factors influencing the virus biology in the context of host–pathogen interaction, focusing on mechanism of action, symptoms severity, therapies, vaccine administration, and role of mutations within new variants [4, 5]. Among factors possibly determining the development of infection and the relative disease severity (as referred to types of symptoms and/or complications), literature has scored the following ones: genetic alterations, comorbidities, age, gender, and microbiome [6–9]. The latter can play a relevant role in the development of many diseases, as well as reported previously [10–15]. Regarding the COVID-19, several papers investigating the relationship between the gut, lung, nasopharyngeal, or oral microbiome and COVID-19 have been published [16–18]. Various mechanisms have been evaluated in several body niches to investigate the interaction between the gut–lung axis and its possible involvement in COVID-19 from different perspectives, such as microbiota, microbiota metabolites, microbial dysbiosis, and mucosal immunity [19–24]. In this regard, our research group has previously described a different microbial composition in nasopharyngeal swabs of patients positive to SARS-CoV-2 respect to healthy subjects [9]. Considering the presence of a niche-specific microbiome within nose and nasopharynx [25], in the present study we analyzed the microbiome of the former using nasal swabs of positive SARS-CoV-2 individuals. In this regard we investigated the microbial composition in such a specific district of the upper respiratory tract and its relation with COVID-19.

## Materials And Methods

### Patients and Controls

In this study we included twenty-two subjects divided in to two groups: *n* = 4 controls (CO) (2 females and 2 males) which resulted as negative to the SARS-CoV-2 molecular assay and *n* = 18 symptomatic COVID-19-positive patients (7 females and 11 males). All the enrolled subjects underwent a nasal swab, performed by a trained otolaryngologist, at admission within the Department of Infectious Diseases at Teaching Hospital of Naples Federico II of Naples, Italy. The inclusion criteria provided that each patient was not being treated with antibiotics, pro- and pre-biotics, antiviral, or corticosteroid medications, for at least one month prior to sample collection. The clinicians evaluated the severity of the symptoms according to the Clinical Status Ordinal Scale as reported by Beigel et al. (2020) [26]. Accordingly, in the cohort of Covid-19 patients there were 3 asymptomatic, 2 showing mild complications, while 12 had mild pneumonia and 1 with severe pneumonia. All of them reported at least loss or change in the sense of smell. In S1 Tab general and clinical characteristics of all COVID-19-positive patients are reported. Moreover, among all COVID-19-positive patients, there were 3 suffering from diabetes, 6 had hypertension, and 1 had cancer. The last was excluded from data analysis being the only one with this clinical condition.

The molecular assay for SARS-CoV-2 was performed within the COVID-19 reference Lab n. 777,777 of CEINGE Biotecnologie Avanzate S.C.a R.L., belonging to the CORONET Campania Regional network for SARS-CoV-2 diagnostics as described previously [9, 27].

### Sample Collection and Bacterial DNA Isolation

We collected a nasal swab from each individual using sterile cotton swabs (COPAN SPA, Brescia, Italy). Bacterial DNA was isolated from each nasal swab sample using MagPurix® Bacterial DNA Extraction Kit (Zinexts Life Science, New Taipei City, Taiwan), according to manufacturer instructions. Qubit dsDNA HS (Severe Sensitivity) assay kit (Invitrogen Co., Life Sciences, Carlsbad, USA) and the TapeStation (Agilent Technologies, Santa Clara, CA, USA) were used to evaluate the yield and the quality of the extracted DNA.

### 16S rRNA Sequencing

Microbiota solution A (Arrow Diagnostics, Genova, Italy) was used to analyze the hypervariable V1–V3 regions of the bacterial 16S rRNA according to the manufacturer instructions and as we reported previously [9]. In particular, we used 2 ng of bacterial DNA as input for the PCR target. The quality and quantity of the amplification products were checked by TapeStation system and Qubit dsDNA HS assay in order to obtain finally an equimolar pool. The sequencing was performed on MiSeq Illumina® sequencing platform (Illumina, CA, US) using V2 500 cycles reagent. We load a pool concentrated to 3 pM and 10% Phix.

To avoid contaminations we performed all steps, from sample collection to library preparation and sequencing, following the precise procedures as detailed previously [11]. Furthermore, to verify the sequencing procedures we also used two standard controls: Oral Microbiome genomic Mix—ATCC MSA-1004 and Gut Microbiome genomic Mix ATCC MSA-1006 (LGC Standard, Milan—Italy) processed simultaneously with the patient samples. The results obtained showed that in these controls the species included in the mix were identified with only slight differences in percentage terms due to the sequencing method compared to the data sheet provided by ATCC.

### Data Analysis

Sequencing data (Fastq files) were analyzed by dedicated bioinformatics software (MicrobAT Suite—SmartSeq, Novara, Italy) that identified the operational taxonomic units (OTUs). As we have done previously, we used MicrobiomeAnalyst program (https://www.microbiomeanalyst.ca/) to perform the statistical analyses, using default parameters. MicrobiomeAnalyst comprises four modules, and we used the Marker Data Profiling (MDP) module that is designed for analysis of 16S rRNA marker gene survey data. The alpha diversity was measured using Chao1, Shannon, and Simpson indices. Mann–Whitney test was performed to calculate the significance of pairwise richness differences. To measure the microbial diversity among the groups, we measured the beta-diversity using Principal Component Analysis (PCoA) using Bray–Curtis dissimilarity index. PERMANOVA (Adonis), Anosim, and PERMDISP2 (Beta-dispersion) tests were used to measure the level of similarity between groups. PERMANOVA tests if the centroids, similar to means, of each group are significantly different from each other. ANOSIM is a method that tests whether two or more groups of samples are significantly different (similar to Adonis). It provides a measure of similarity and its statistic R is based on the difference of mean ranks between groups and within groups. Having both significant tests gives more strength to the hypothesis of different composition between groups. PERMDISP is a measure of dispersion (variances) of the groups. If significant, the two groups are not homogeneously dispersed. PERMANOVA and PERMDISP can be used to rigorously identify location *versus* dispersion effects, respectively, in the space of the chosen resemblance measure.

Abundances of taxa were evaluated in HC and COVID positive patients using Classical Univariate Statistical Comparisons (ANOVA test) available in Microbiomeanalyst suite at each taxonomic level.

Two-sample Wilcoxon rank-sum (Mann–Whitney), nonparametric equality of medians tests and the Spearman’s correlation was performed using STATA, Statistical Software Release 12. The raw and processed high-throughput sequencing data have been deposited in the Sequence Read Archive (SRA) (https://www.ncbi.nlm.nih. gov/sra) under Project PRJNA744167.

## Results

**Fig. 1:**
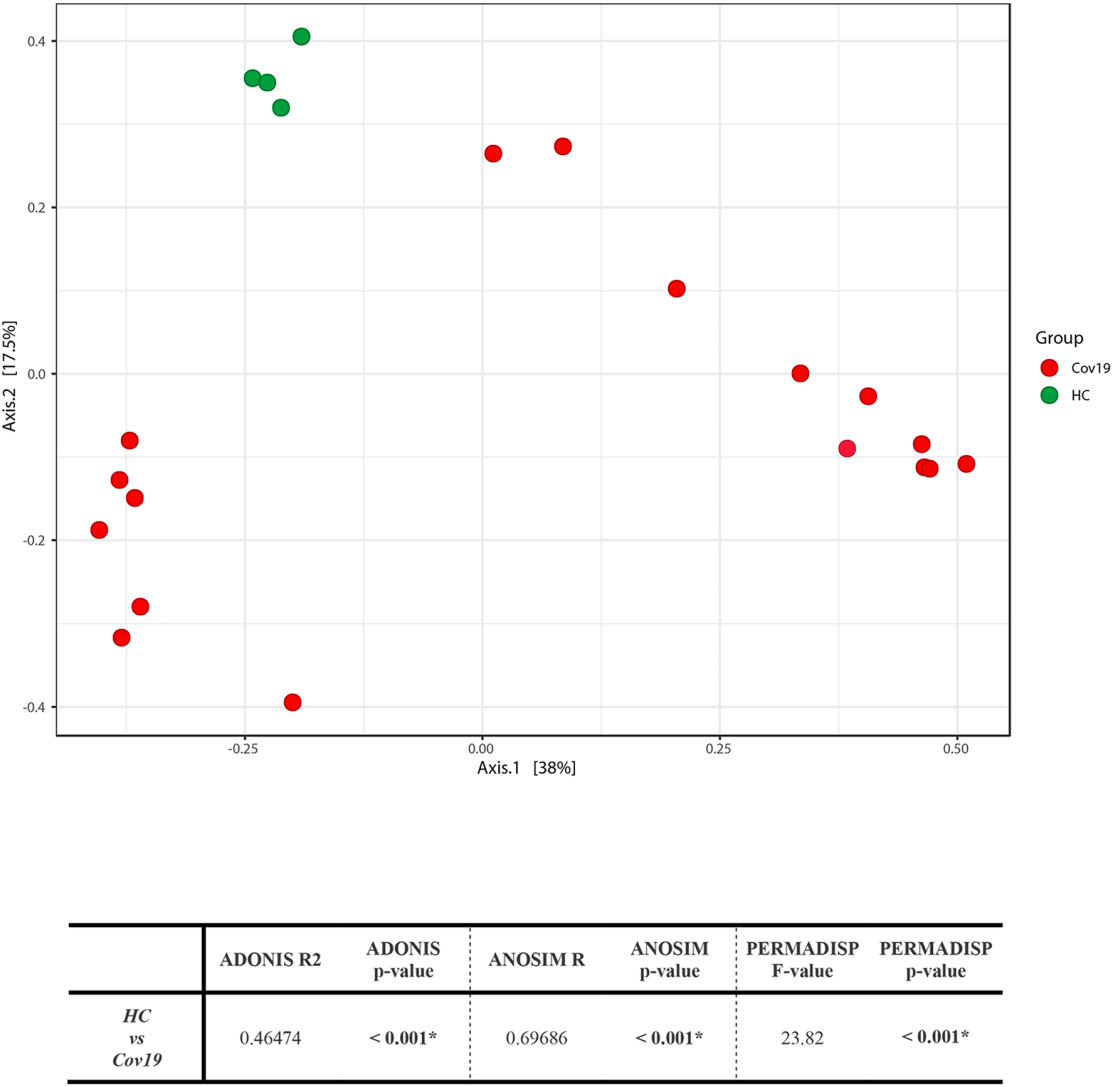
Beta-diversity of bacteria identified in the COVID-19 (Cov19) and Healthy Control (HC) groups. Principal Component Analysis (PCoA) plots using the Bray–Curtis distance measures. Results of PERMANOVA (ADONIS), ANOSIM, and PERMADISP tests shown in the table (HC vs Cov19) indicated both a significant separation between the centroids and differences between groups (*P*< 0.001). Red and green blots indicated Cov19 patients and HC subjects, respectively (Color figure online)

The sequencing of all nasal swabs produced a total of 447,558 counts. The alpha diversity, the measure of within-sample diversity, and the comparison of the species richness among the analyzed groups, through Chao1, Shannon, and Simpson indices, showed significantly increased microbial richness in Cov19 patients compared to HC (Chao1: *P* = 0.001, Shannon: *P* = 0.007, Simpson: *P* = 0.009). Beta-diversity, the measure of the dissimilarities among groups, was evaluated via the PCoA using Bray–Curtis index, showing a different microbial composition between the analyzed groups (*P* = 0.002) (Fig. 1). Interestingly, we clearly identified two clusters, associated with gender of the patients, within the Cov19 group as reported in S1 Fig, but in this study we did not focus our analysis on these clusters.

**Fig. 2:**
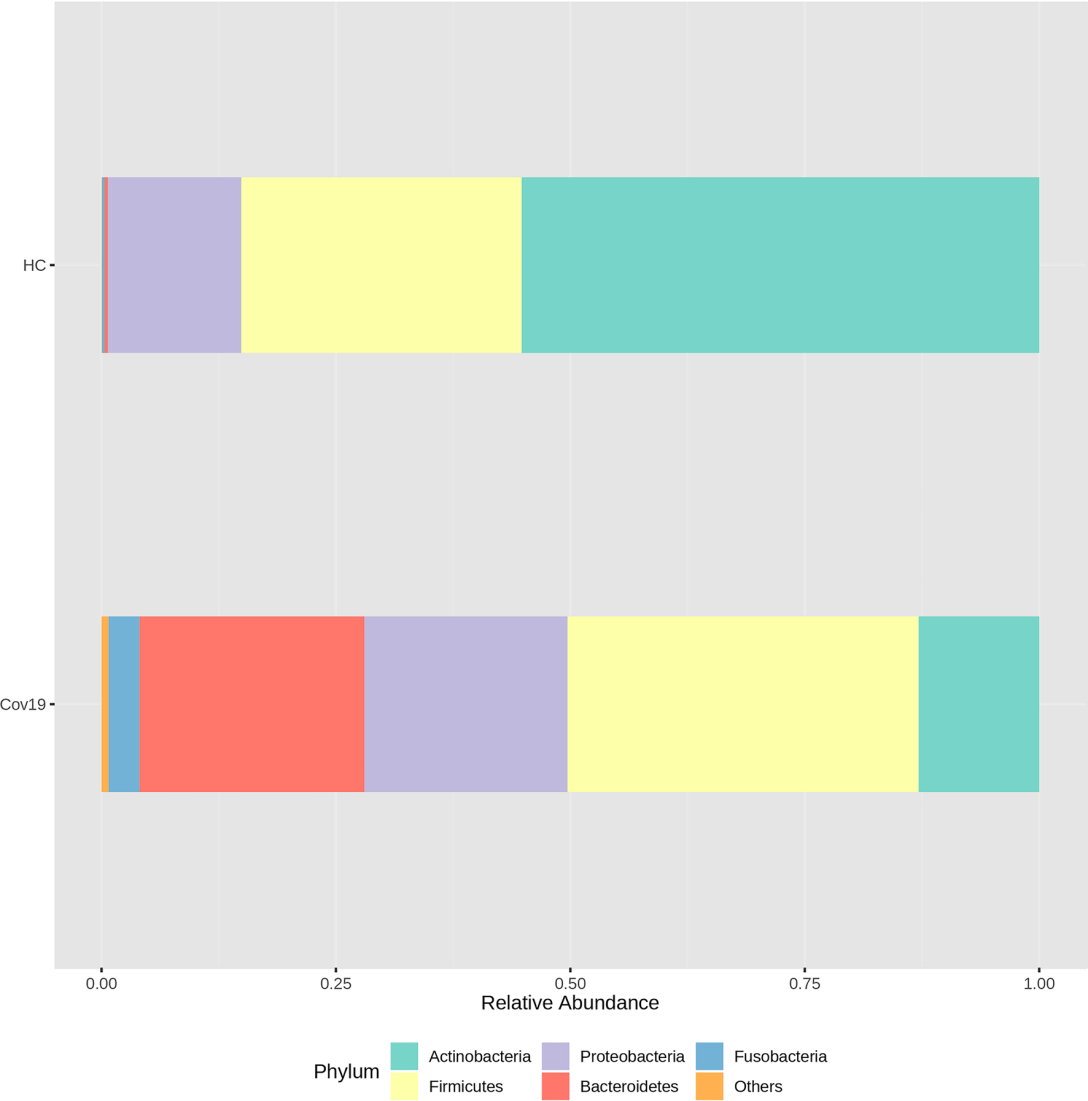
Abundance Profiling of nasal microbiome composition in Cov19 and HC groups. The stacked bars showed the top 5 phyla in the HC and Cov19 groups as identified by the MicrobAT Suite–SmartSeq. Color and width of the column denoted the relative abundance (%) for each phylum. Actinobacteria abundance was significantly reduced, by ANOVA, in Cov19 patients, with respect to HC group (*P* < 0.001). Not statistically significant differences were observed comparing the abundance of other phyla among the groups. Current figure was downloaded by https://www.microbiomeanalyst.ca/MicrobiomeAnalyst/home.xhtml after our data analysis (Color figure online)

The measure of the beta-diversity showed that the nasal microbiome in patients with COVID-19 was different from that of healthy subjects (HC), even if this latter group was very small. To assess the reproducibility of our results, we compared the relative abundances of the microbial community in the nasal cavity of our control group with those characterized in 12 healthy subjects by Bassis et al. [28] (PRJNA248297). Four subjects have been excluded from the analysis as the ethnicity of two samples was not Caucasian (called “white”), namely subject A and H, while for subject K and L sequencing data were not available. In total 8 healthy individuals (6 females and 2 males) showing an average age of 38.8 ± 10.8 were selected. To assess that the microbial composition of our HC group was similar to that found by Bassis et al. (PRJNA248297 BioProject), we performed both two-sample Wilcoxon rank-sum (Mann–Whitney) and nonparametric equality of medians tests using STATA. Firstly, we collected the supplementary data from Bassis et al. showing the relative abundances at family level. Four samples were filtered out as described above and then we evaluated if the relative abundances in control group A (Nardelli et al.) and control B (Bassis et al.) were superimposable by means of nonparametric tests. As reported in S2 Tab, we found that all nasal microbial families were not statistically different in terms of abundances, demonstrating that our control group, even if small, was very similar to the healthy samples analyzed by Bassis et *al*. There was only a slight difference at the level of Fusobacteriaceae, Streptococcaceae and Burkholderiales incertae sedis. Consequently, we compared the relative abundances at family level for all the available control samples (A + B) against Cov19 samples and we observed a strong significant difference (Mann–Whitney, *P* = 0.0004) at Corynebacteriaceae level (Actinobacteria). Moreover, other microbial communities (Propionibacteriaceae, Porphyromonadaceae, Burkholderiales incertae sedis, Bacillales Incertae Sedis XI, Clostridiales Incertae Sedis XI, Staphylococcaceae, Veillonellaceae, Moraxellaceae) were found differentially abundant, even if a lesser extent compared to the Corynebacteriaceae family (S2 Tab). Once assuming that the data from Bassis et al. overlap our control dataset, even if small, we were more confident in the interpretation of our findings. Indeed, we found that the main taxa identified in nasal microbiome of Cov19 patients and in HC subjects belonged to three distinct phyla: Proteobacteria (HC = 14%, Cov19 = 35.8%), Firmicutes (HC = 28.8%, Cov19 = 30.6%), and Actinobacteria (HC = 56.7%, Cov19 = 14.4%) with a relative abundance > 1% in all groups. In addition, we also showed the Bacteroidetes and Fusobacteria because, despite having an abundance < 1% for HC group, showed a positive trend from HC to Cov19 patients (Fig. 2 and Table. 1). The comparison of the relative abundances between two groups showed that the Actinobacteria abundance was significantly reduced in Cov19 patients, with respect to HC group (*P* < 0.001), while not statistically significant differences were observed for other phyla (Table. 1).

**Table 1:** Nasal microbiome composition at phylum level (excluding unclassified taxa) in the Cov19 patients and healthy control group

Phylum	*Cov19 (%)*	*HC (%)*	ANOVA (*P*-value)	FDR
Proteobacteria	*35.8*	*14.0*	0.23	0.41
Firmicutes	*30.6*	*28.8*	0.78	0.91
Bacteroidetes	*16.1*	*0.2*	0.08	0.28
Actinobacteria	*14.4*	*56.7*	** < 0.001**	** < 0.01**
Fusobacteria	*2.6*	*0.2*	0.16	0.38
Others	*0.5*	*0.0*	ns	ns

Abundances of phyla were evaluated in HC and Cov19-positive patients using Classical Univariate Statistical Comparisons (ANOVA test using the Mann–Whitney statistical method). Statistically significant values were reported in bold

**Fig. 3:**
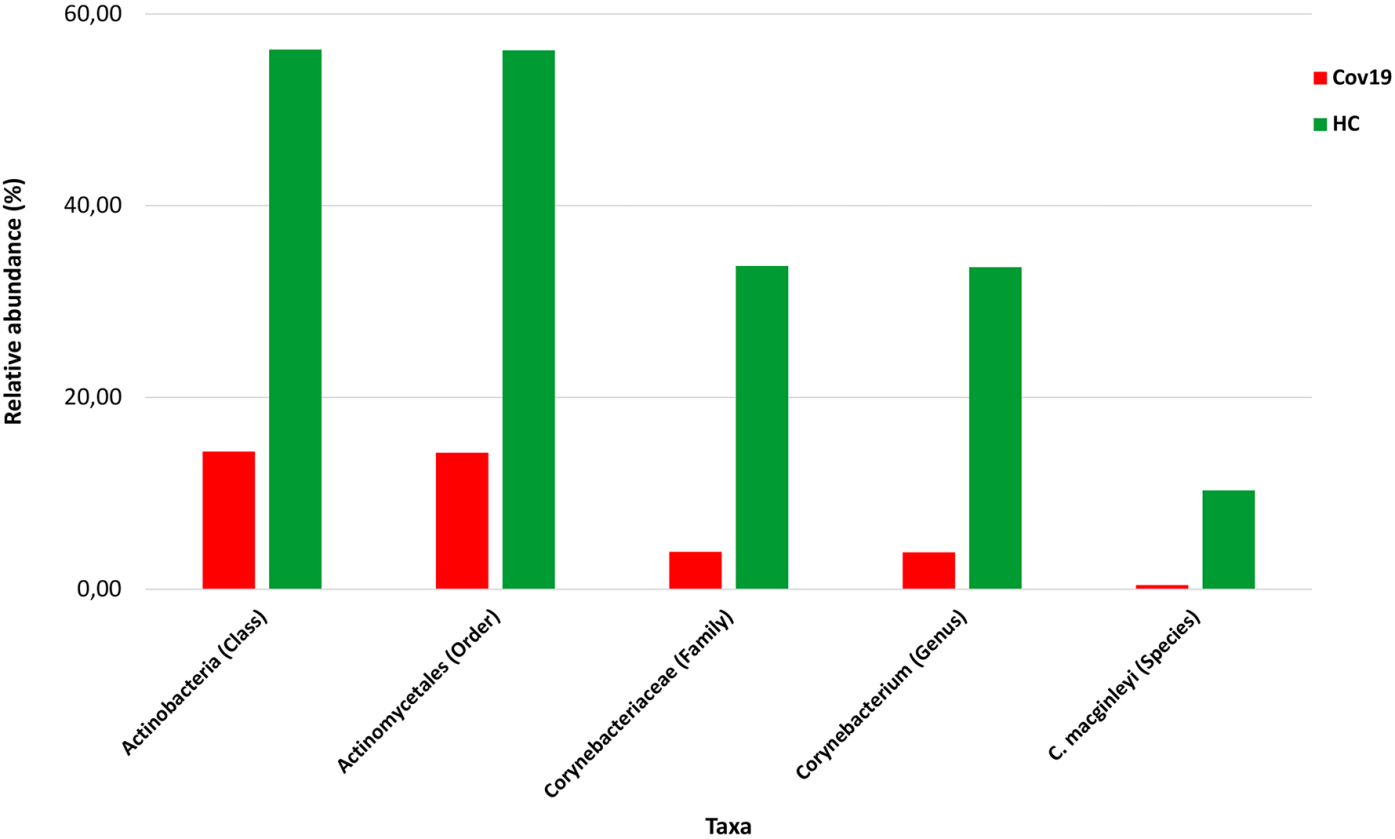
Negative trend of the Actinobacteria (from the class down to the species) in the nasal swabs of Cov19 patients compared to HC group. The bar plots showed the average relative abundance (%) of the Actinobacteria from the class to the species. Differences were statistically significant between groups using the ANOVA test (*P* < 0.01). Bars were color coded according to each group: red for Cov19 and green for HC (Color figure online)

The significant reduction of Actinobacteria between groups was evaluated from the class up to the species (*P* < 0.01) using the ANOVA test (Fig. 3). The picture showed the average relative abundance (%) of the Actinobacteria through several taxonomic levels in which differences were always statistically significant (*P* < 0.01).

**Fig. 4:**
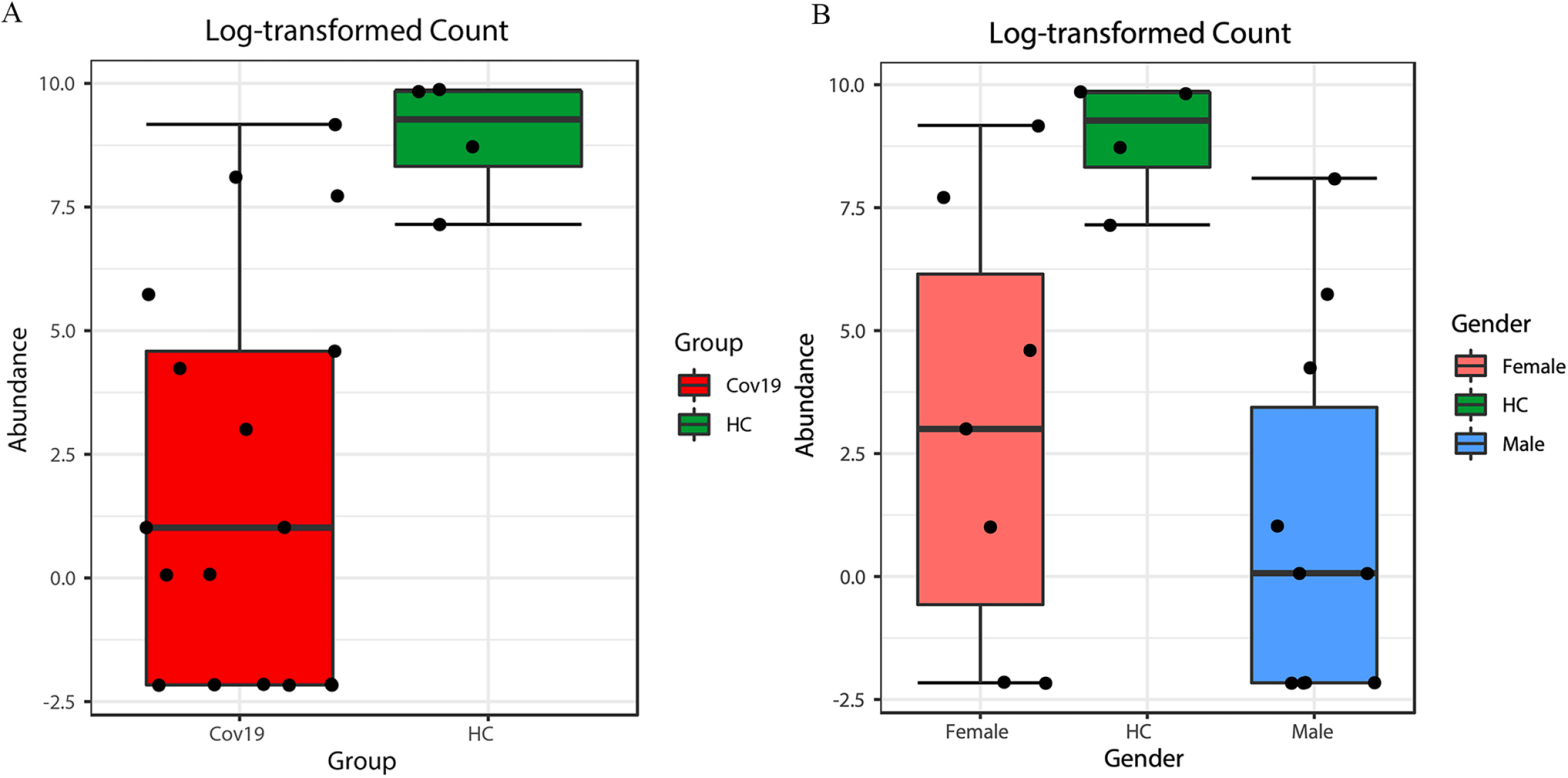
Relative abundance of Corynebacterium in HC versus Cov19 (**A**) and in female and male Cov19 subgroups (**B**). Box plot showed in (**A**) the log-transformed count of Corynebacterium in Cov19 (red box) versus HC (green box) (*P* = 0.004) and (**B**) the relative abundance of Corynebacterium in Cov19 group split in to Female (pink box) and Male (blue box) (their comparison was not significant), compared to HC (green box) (Color figure online)

Consequently, to confirm that the *Corynebacterium* relative abundance was not significantly different within the two clusters identified in S1 Fig (Cov19 gender related), female and male subgroups were investigated performing an univariate analysis at the genus level. We did not further investigate the differences found as these were not included as primary outcomes of this study. Nevertheless, since there were no significant differences in clinical and biochemical parameters (S3 Tab) between females and males, except for weight, height, and fibrinogen level, we did not correlate them with the bacterial community. Regardless, we performed Spearman’s correlation of *Corynebacterium* abundance with all available features. To note, we did not spot any correlation in the female group, while in males *Corynebacterium* correlated with platelets (*r* = − 0.7153, *P* = 0.013) and alanine aminotransferase (*r* = − 0.638, *P* = 0.0347). In Fig. 4, the box plot reported the log-transformed count of the Cov19 versus HC for the *Corynebacterium* (panel A). We further split the Cov19 group by gender (panel B), and we did not observe any significant difference at this level.

## Discussion

The present pilot study described the nasal microbiome changing in Cov19 patients compared to not-affected subjects. Interestingly, the nasal microbial composition resulted different between the study groups, as shown by the beta-diversity. Furthermore, our data showed a significantly increased microbial diversity in affected individuals compared to healthy subjects, as already reported in similar settings [29–31]. Noteworthy, we found a relevant reduced abundance of the Actinobacteria in Cov19 patients as compared to controls, and this difference was observable until species level [Actinobacteteria (class), Actinomycetales (order), Corynebacteriaceae (family), *Corynebacterium* (genus), and *C. macginleyi* (species)].

By evaluating the clinical features of our study group, we observed in all Cov19 patients the presence of a common symptom, the loss of smell. Consequently, focusing on the *Corynebacterium* genus, we hypothesized a possible relationship between the reduced abundance of this taxon and anosmia in Cov19 patients. The loss of smell was a common symptom associated with SARS-CoV-2 infection caused by several factors determining both acute and chronic olfactory dysfunctions [32]. In fact, the epithelium of the respiratory system was the primary site of coronavirus attachment and so the viral impacts on the sense of smell and taste have not been surprising. Among the mechanisms suggested to cause anosmia in COVID-19 [33], the inflammatory pattern following the SARS-CoV-2 infection in the nasal cavity could represent a possible additive mechanism triggering the olfactory dysfunction, also exacerbated by the dysbiosis [29–31]. In this regard, *Corynebacterium* was known to be reduced in individuals with loss of smell, even in the absence of SARS-CoV-2 infection [32]. More recently, the reduced abundance of this bacterium has also been associated with the severity of COVID-19 symptoms [34].

Even though our control group was very small, we confirmed data published by De Boeck et al. [25] which showed *Corynebacterium* as highly represented in nasal swabs of healthy individuals. Besides, to reinforce our data, we compared the HC group’s microbial profile with one described in healthy subjects by Bassis et al., showing a similar abundance of Actinobacteria [28].

In keeping with our data, we underlined that also the findings by Ngo et al. [35] indicated the microbiota as a potentially modifiable factor influencing COVID-19. Indeed, the latter authors reviewed the literature drawing conclusions about the plethora of potential means which supported the microbiota of gut and respiratory tract in COVID-19 onset and its related consequences [35]. Nevertheless, the mechanisms surrounding these effects were far from being completely elucidated. To our knowledge no data were reported about the relationship of nasal microbiome and anosmia in patients with COVID-19.

In addition to that, Koskinen et al. found increased diversity in subjects with partial or complete smell impediments compared to normosmic participants [30]. This was usually considered as a positive feature, but in microbial nasal composition this condition could have a different impact. The authors found butyric acid-producing microorganisms associated with impaired olfactory function [30]. We would also underline as Kumpitsch et al. reported that such Firmicutes species, capable of producing butyrate, could impact on olfactory performance [31]. The butyrate had a very strong and unpleasant odor and its production was out of place in the nasal area; therefore, to improve the quality of life, authors proposed to support patients suffering from olfactory dysfunction with probiotics [31]. According to that, we found in our patients an increased amount of OTU associated to Firmicutes phylum respect to healthy subjects, even if not at a significant level.

Our evidence was also supported by the literature and thus we can speculate that SARS-Cov-2 could play a role in changing the protective microbiome film of nasal mucosa, increasing the quantity of species able to produce butyrate and giving olfactory dysfunctions, like loss of smell.

## Conclusion

In our study, we are not able to establish who drives who, but this incidental finding could open a new field of investigation in the setting of potential interactions between SARS-Cov-2 and microbiome milieu. We highlight how the *Corynebacterium* could act as a potential biomarker linked to the loss of olfactory function in COVID-19 patients. Reasonably, we are conscious that further studies are required to understand the mechanisms underlying the loss of olfactory function that we herein associate with the reduction of *Corynebacterium* in COVID-19 patients. We are aware of the small cohort enrolled in the study and so we were unable to draw general conclusions in differentiating the composition among HC and Cov19, as well as female and male. Nevertheless, we would underline the difficulty in enrolling “naïve” patients without evident confounding factors influencing the nasal microbiome content, particularly during the COVID-19 pandemic spread. Here we present the data gained from our pilot study aiming to give a first insight into the nasal microbiota of COVID-19 patients. As a matter of course, further studies are required.

## Supplementary Information

Below is the link to the electronic supplementary material.

Supplementary file1 (TIF 6579 kb)
S1 Fig. Beta-diversity of bacteria identified in the COVID-19 patients divided by gender (Female and Male) and Healthy Control (HC) groups. Principal Component Analysis (PCoA) plots using the Bray–Curtis distance measures. Results of PERMANOVA (ADONIS), ANOSIM, and PERMADISP tests showed significant separation between all the centroids and differences between groups (p<0.001). Green dots indicate HC subjects; pink and blue dots indicate Female and Male patients, respectively.

Supplementary file2 (XLSX 15 kb)

Supplementary file3 (DOCX 21 kb)

Supplementary file4 (DOCX 18 kb)

## Data Availability

The datasets for this study can be found in the GenBank Database PRJNA744167 at the following link: https://dataview.ncbi.nlm.nih.gov/object/PRJNA744167?reviewer=r10098eahc2rnf80socjomnr4q
