# Supplementary figures and images for: Nasal Microbiome in COVID-19: A Potential Role of Corynebacterium in Anosmia
Source: Curr Microbiol. 2022 Dec 30;80(1):53. doi: 10.1007/s00284-022-03106-x (PMC9802018; doi:10.1007/s00284-022-03106-x)

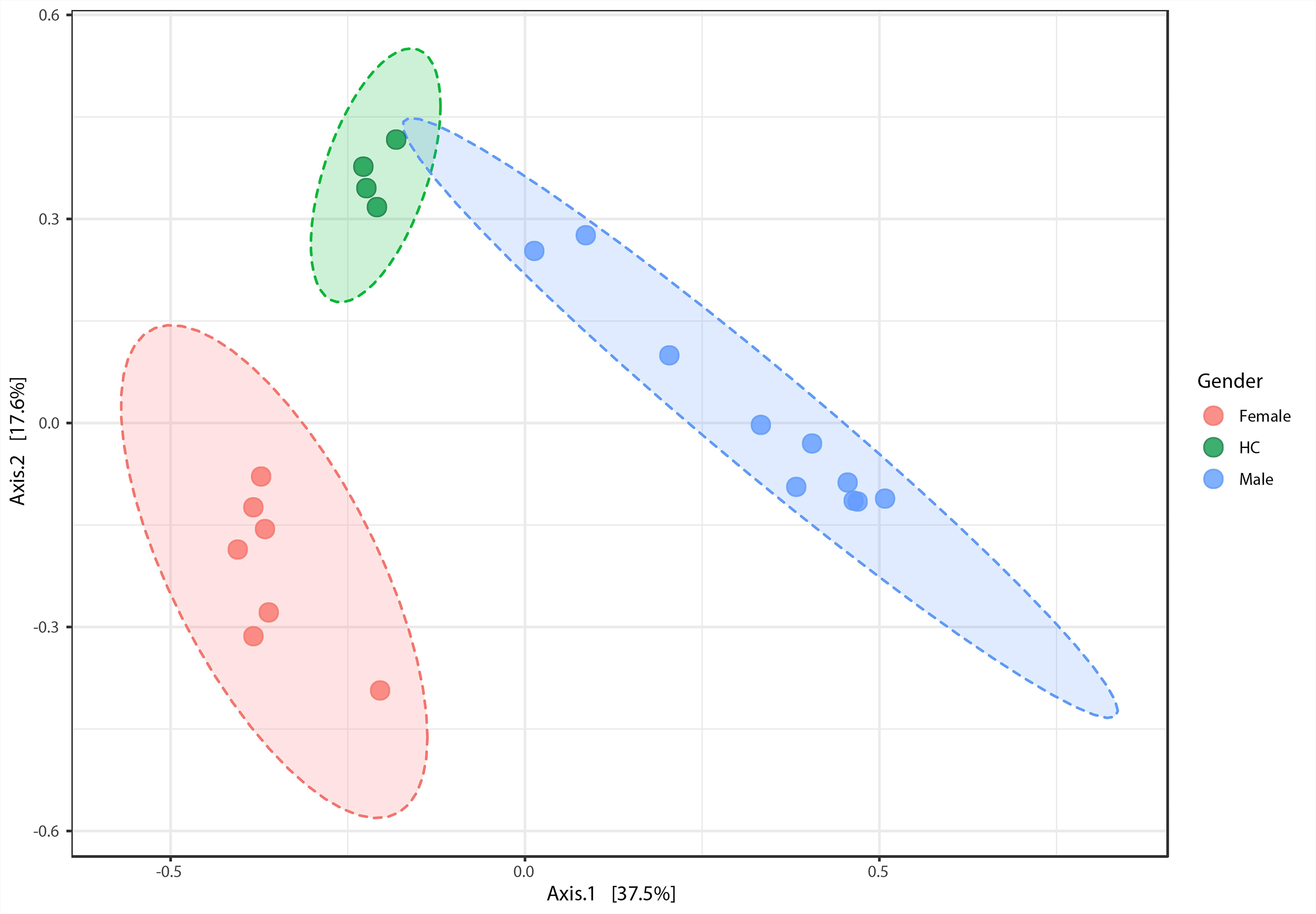

Supplement: Supplementary file 1 — Supplementary file1 (TIF 6579 kb) S1 Fig. Beta-diversity of bacteria identified in the COVID-19 patients divided by gender (Female and Male) and Healthy Control (HC) groups. Principal Component Analysis (PCoA) plots using the Bray–Curtis distance measures. Results of PERMANOVA (ADONIS), ANOSIM, and PERMADISP tests showed significant separation between all the centroids and differences between groups (p<0.001). Green dots indicate HC subjects; pink and blue dots indicate Female and Male patients, respectively. [file 284_2022_3106_MOESM1_ESM.tif]
