# Supplementary material for: Nasal Microbiome in COVID-19: A Potential Role of Corynebacterium in Anosmia
Source: Curr Microbiol. 2022 Dec 30;80(1):53. doi: 10.1007/s00284-022-03106-x (PMC9802018; doi:10.1007/s00284-022-03106-x)
Supplement: Supplementary file 3 — Supplementary file3 (DOCX 21 kb) [file 284_2022_3106_MOESM3_ESM.docx]

***Nasal microbiome in COVID-19: a potential role of Corynebacterium in anosmia***

*Carmela Nardelli§, Giovanni Luca Scaglione§, Domenico Testa, Mario Setaro, Filippo Russo, Carmela Di Domenico, Lidia Atripaldi, Massimo Zollo, Federica Corrado, Paola Salvatore, Biagio Pinchera, Ivan Gentile, Ettore Capoluongo**

**S2 Tab.** Non parametric tests performed between control groups A & B. Group A: n=4 healthy subjects from this study; Group B: n=8 healthy samples from Bassis et *al*. For all the microbial families available in both studies, Mann-Whitney and median tests were performed to: a) check that the control groups have similar relative abundances (p<0.05); b) the merged/extended control group data show the expected significant differences compared to the Cov19 group.

| **Phylum** | **Family** | **Control group A (Nardelli *et al)*** | | | | **HC (merged)** | |
| --- | --- | --- | --- | --- | --- | --- | --- |
|  |  | ***vs*** | | | | ***vs*** | |
|  |  | **Control group B (Bassis et *al)*** | | | | **Cov19** | |
|  |  | Median  (5th-95th percentile) | | Mann-Whitney  (p-value) | nonparametric  k-sample test  (p-value) | Mann-Whitney  (p-value) | nonparametric  k-sample test  (p-value) |
|  |  | **Control A** | **Control B** |  |  |  |  |
| Actinobacteria | Actinomycetaceae | 0.05 (0-0.4) | 0.4 (0-2.3) | ns | ns | ns | ns |
|  | **Corynebacteriaceae** | 38.1 (10.2-55.9) | 9.45 (2.3-63.1) | ns | ns | **0.0004** | **0.0009** |
|  | Micrococcaceae | 0.2 (0.1-0.4) | 0 (0-0.6) | ns | ns | ns | ns |
|  | Propionibacteriaceae | 14.05 (7.2-23.4) | 13.95 (0.8-35) | ns | ns | **<0.05** | **<0.05** |
| Bacteroidetes | Flavobacteriaceae | 0.05 (0-0.6) | 0.55 (0-2.7) | ns | ns | ns | ns |
|  | Porphyromonadaceae | 0.15 (0.1-0.3) | 0.7 (0-2.9) | ns | ns | **<0.05** | **<0.05** |
|  | Prevotellaceae | 0.15 (0.1-0.3) | 0.7 (0-2.9) | ns | ns | ns | ns |
|  | Burkholderiales incertae sedis | 0.05 (0-0.1) | 0.55 (0.1-4.9) | **0.024** | ns | **<0.05** | ns |
|  | Comamonadaceae | 0.75 (0.4-3.4) | 1.3 (0-7.8) | ns | ns | ns | ns |
|  | Neisseriaceae | 0.3 (0-1.4) | 0.35 (0-12.7) | ns | ns | ns | ns |
| Firmicutes | Bacillales Incertae Sedis XI | 0 (0-0) | 0 (0-1) | ns | ns | **<0.05** | **<0.05** |
|  | Carnobacteriaceae | 0.05 (0-0.1) | 0.2 (0-12) | ns | ns | ns | ns |
|  | Clostridiales Incertae Sedis XI | 2.4 (0.6-21.3) | 2.05 (0-4.3) | ns | ns | **<0.05** | **<0.05** |
|  | Lactobacillaceae | 0 (0-0.1) | 0 (0-0.9) | ns | ns | ns | ns |
|  | Peptostreptococcaceae | 0 (0-0.1) | 0 (0-12.8) | ns | ns | ns | ns |
|  | Staphylococcaceae | 16.1 (6.7-38.3) | 9.7 (2.6-52.4) | ns | ns | **<0.05** | **<0.05** |
|  | Streptococcaceae | 0.25 (0.1-1.3) | 5 (0.8-44.3) | **0.02** | ns | ns | ns |
|  | unclassified Lactobacillales | 0 (0-0) | 0.7 (0-1.6) | ns | ns | ns | ns |
|  | Veillonellaceae | 0.1 (0-0.1) | 0.7 (0-3.1) | ns | ns | **<0.05** | ns |
| Fusobacteria | Fusobacteriaceae | 0.15 (0.1-0.5) | 0 (0-0.3) | **0.013** | **0.02** | ns | ns |
|  | Leptotrichiaceae | 0 (0-0.9) | 0 (0-0.7) | ns | ns | ns | ns |
| Gamma-proteobacteria | Moraxellaceae | 0.2 (0.1-0.4) | 0.4 (0-3.3) | ns | ns | **0.0532** | ns |
|  | Gamma-proteobacteria _other | - | 0.2 (0-1.4) | NA | NA | NA | NA |
|  | Pasteurellaceae | 0.05 (0-0.1) | 0.2 (0-1.9) | ns | ns | ns | ns |

**ns = not significant; NA = not available**
