# Supplementary material for: Nasal Microbiome in COVID-19: A Potential Role of Corynebacterium in Anosmia
Source: Curr Microbiol. 2022 Dec 30;80(1):53. doi: 10.1007/s00284-022-03106-x (PMC9802018; doi:10.1007/s00284-022-03106-x)
Supplement: Supplementary file 4 — Supplementary file4 (DOCX 18 kb) [file 284_2022_3106_MOESM4_ESM.docx]

***Nasal microbiome in COVID-19: a potential role of Corynebacterium in anosmia***

*Carmela Nardelli§, Giovanni Luca Scaglione§, Domenico Testa, Mario Setaro, Filippo Russo, Carmela Di Domenico, Lidia Atripaldi, Massimo Zollo, Federica Corrado, Paola Salvatore, Biagio Pinchera, Ivan Gentile, Ettore Capoluongo**

**S3 Tab.** General and clinical characteristics of patients with Cov19 divided by gender.

| **Parameters** | **Females (n=7)** | | **Males (n=11)** | |
| --- | --- | --- | --- | --- |
|  | **Mean** | **SD** | **Mean** | **SD** |
| **Age (years)** | **39.1** | **21.4** | **53.0** | **13.8** |
| **Weight (Kg) *** | **70.9** | **7.2** | **78.6** | **5.9** |
| **Height (m) *** | **1.70** | **0.05** | **1.80** | **0.04** |
| **BMI (Kg/m^2^)** | **24.6** | **2.8** | **25.4** | **1.8** |
| **Smokers** | **2** | **-** | **9** | **-** |
| **Red blood cells (U/L)** | **4834285** | **503847** | **4375091** | **1360616** |
| **White blood cells (U/mL)** | **8967** | **2592** | **7715** | **1158** |
| **Neutrophils (U/mL)** | **7023** | **2260** | **6259** | **1191** |
| **Lymphocytes (U/mL)** | **1227** | **317** | **1635** | **1661** |
| **Hemoglobin (g/mL)** | **11.40** | **1.64** | **12.28** | **0.90** |
| **Platelets (U/mL)** | **293857** | **87659** | **308526** | **33302** |
| **Lactate Dehydrogenase (U/L)** | **357.0** | **116.5** | **432.1** | **89.9** |
| **Aspartate aminotransferase (U/L)** | **27.6** | **8.7** | **35.7** | **11.4** |
| **Alanine aminotransferase (U/L)** | **28.0** | **5.4** | **35.0** | **14.2** |
| **Creatinine (mg/dL)** | **0.9** | **0.2** | **0.9** | **0.1** |
| **Azotemia (mg/dL)** | **38.9** | **13.7** | **48.5** | **13.0** |
| **Creatine Phosphokinase (U/L)** | **57.0** | **19.3** | **76.0** | **23.0** |
| **Prothrombin time (s)** | **0.95** | **0.10** | **0.93** | **0.10** |
| **D-Dimer (ng/mL)** | **1.2** | **0.6** | **1.3** | **0.3** |
| **Fibrinogen (mg/mL) **** | **449** | **81** | **571** | **59** |
| **Reactive C protein (mg/L)** | **7.5** | **3.6** | **9.2** | **3.2** |
| *** p<0.05; ** p<0.001** |  |  |  |  |
